# Liposomes Loaded with Amaranth Unsaponifiable Matter and Soybean Lunasin Prevented Melanoma Tumor Development Overexpressing Caspase-3 in an In Vivo Model

**DOI:** 10.3390/pharmaceutics14102214

**Published:** 2022-10-18

**Authors:** Erick Damian Castañeda-Reyes, María de Jesús Perea-Flores, Gloria Dávila-Ortiz, Elvira Gonzalez de Mejia

**Affiliations:** 1 Vegetal Proteins Laboratoy, Department of Biochemical Engineering, National School of Biological Sciences, National Polytechnic Institute, (IPN), Av. Wilfrido Massieu, and Miguel Stampa s/n, Zacatenco, Gustavo A. Madero, Mexico City 07738, Mexico; 2 National Center of Nanosciences and Micro and Nanotechnologies, National Polytechnic Institute (IPN), Av. Luis Enrique Erro s/n, Unidad Profesional Adolfo López Mateos, Zacatenco, Alcaldía Gustavo A. Madero, Mexico City 07738, Mexico; 3 Department of Food Science and Human Nutrition, 228 ER Madigan Laboratory, University of Illinois, 2101 W. Gregory Dr, Champaign, IL 61801, USA

**Keywords:** melanoma, liposomes, soybean lunasin, squalene from amaranth (*Amaranthus hypochondriacus*), in vivo model, C57BL/6 mice

## Abstract

The objective of this study was to assess the effectiveness of liposomes loaded with soybean lunasin and amaranth unsaponifiable matter (UM + LunLip) as a source of squalene in the prevention of melanoma skin cancer in an allograft mice model. Tumors were induced by transplanting melanoma B16-F10 cells into the mice. The most effective treatments were those including UM + LunLip, with no difference between the lunasin concentrations (15 or 30 mg/kg body weight); however, these treatments were statistically different from the tumor-bearing untreated control (G3) (*p* < 0.05). The groups treated with topical application showed significant inhibition (68%, *p* < 0.05) compared to G3. The groups treated with subcutaneous injections showed significant inhibition (up to 99%, *p* < 0.05) in G3. During tumor development, UM + LunLip treatments under-expressed Ki-67 (0.2-fold compared to G3), glycogen synthase kinase-3β (0.1-fold compared to G3), and overexpressed caspase-3 (30-fold compared to G3). In addition, larger tumors showed larger necrotic areas (38% with respect to the total tumor) (*p* < 0.0001). In conclusion, the UM + LunLip treatment was effective when applied either subcutaneously or topically in the melanoma tumor-developing groups, as it slowed down cell proliferation and activated apoptosis.

## 1. Introduction

Melanoma is the third most dangerous skin cancer [1]; it has a 5-year survival rate of 5–20% [2]. The American Cancer Society [3] estimated the diagnosis of approximately 99,780 new melanoma cases in 2022; approximately 7659 people are expected to die of melanoma. The rates of melanoma vary with age and have increased rapidly over the past few decades.

The most common somatic mutation involved in melanoma is the B-raf proto-oncogene (BRAF). The oncogenic constitutive activation of the BRAF kinase through mutation accounts for approximately 40–60% of melanoma cases, with 90% of these being V600E mutations (a mutation in codon 600 of exon 15 of the BRAF gene), characterized by the substitution of valine for glutamic acid at codon 600 [4,5]. Some melanoma treatments include therapies using BRAF, mitogen-activated extracellular signal-regulated kinase, or programmed cell death-1 (PD-1) inhibitors such as vemurafenib or trametinib [6,7]. In contrast, the inhibition of glycogen synthase kinase-3β (GSK-3β) can suppress tumor growth at the same level as the anti-PD-1 drug [6]. GSK-3β is a serine/threonine protein kinase whose normal activity allows cells to maintain homeostasis [8]. There are considerable risks in melanoma drug treatments, including potential drug toxicity, adverse side effects, and drug resistance, that come with currently available treatments [4,7,9]. As a result, the development of an alternative liposome-based treatment is imperative for improving the therapeutic response of melanoma patients [10].

The cell internalization mechanisms of liposomes include clathrin-mediated endocytosis (CME), the caveolae-mediated endocytosis pathway (CvME), and clathrin-/caveolae-independent endocytosis, such as macropinocytosis [11]. Liposomes can encapsulate polar, nonpolar, and amphiphilic substances, enabling the delivery of a wide variety of therapeutics and other bioactive compounds [10]. Liposomes reduce toxicity and drug degradation, given the presence of a lipid bilayer, and reduce drug inactivation, given their carrying capacity [9,12].

Soybean peptide lunasin has been successfully tested against melanoma [13,14,15,16,17]. The anticancer effects of lunasin on chemically induced skin cancer and melanoma tumor growth rates in mice have been reported [13,14,17,18]. Lunasin is a 43-amino acid peptide that has been shown to block cell cycle progression and induce apoptosis while simultaneously preventing cell adhesion and metastasis, which, consequently, prevents tumor formation [19]. Lunasin-loaded liposomes improve lunasin cytotoxicity in A375 and B16-F10 melanoma cells [15].

Another bioactive molecule of interest is squalene, which is used as an adjuvant in vaccines and cancer treatments given its notable anticancer effects, ability to improve the immune response, and capability to increase cell permeability [20,21]. Squalene has also been used in adhesive films composed of chitosan-coated nanostructured lipid carriers that contain simvastatin. It serves as a nanostructuring agent and skin permeation enhancer, increasing uptake into melanoma cells and improving cytotoxicity when compared to free simvastatin [21].

We hypothesized that the soybean peptide lunasin and the amaranth unsaponifiable matter, as a source of squalene, transported into liposomes, effectively prevent melanoma tumor development in an in vivo model of C57BL/6 mice.

Therefore, the objective of this study was to assess the effectiveness of liposomes loaded with the soybean peptide lunasin and amaranth unsaponifiable matter, as a source of squalene, in the prevention of melanoma in a skin cancer allograft model using C57BL/6 mice.

## 2. Materials and Methods

### 2.1. Materials

B16-F10 mouse melanoma cell line (ATCC^®^ CRL6475™) was purchased from the American Type Culture Collection (Manassas, VA, USA). Cytochalasin D, chlorpromazine hydrochloride, genistein, 1,2-dioleoyl-sn-glycero-3-phospho-rac-(1-glycerol) (DOPG), and 1,2-dioleoyl-sn-glycero-3-phosphocholine (DOPC) were purchased from Sigma (St. Louis, MO, USA). Mouse monoclonal anti-glycogen synthase kinase-3β (GSK-3β) (ab93926, epitope within amino acids 292–420); and rabbit monoclonal anti-caspase-3 (ab184787, epitope within amino acids 1–175 recognizing total caspase-3) were purchased from Abcam (Cambridge, MA, USA). In addition, 1,2-dioleoyl-sn-glycero-3-phosphoethanolamine-*N*-(lissamine rhodamine B sulfonyl) was purchased from Avanti Polar Lipids (Alabaster, AL, USA). Ibidi^TM^ µ-Dish 35 mm, high ibiditreat were purchased from Ibidi (Fitchburg, WI, USA). Hoechst 33342, modified Harris Hematoxylin (72711), and eosin (71304) were purchased from Thermo Scientific (Rockford, IL, USA). Rodent Block M, Mouse-on-Mouse HRP-Polymer, Background Sniper, and Rabbit-on-Rodent HRP Polymer were purchased from Biocare Medical, (Pacheco, CA, USA).

### 2.2. Methods

#### 2.2.1. Liposome Preparation

Liposomes were prepared as described in our previous study [15]. Liposomes were prepared using the phospholipids 1,2-dioleoyl-sn-glycero-3-phospho-rac-(1-glycerol) and 1,2-dioleoyl-sn-glycero-3-phosphocholine 1:1 molar ratio; amaranth unsaponifiable matter and phospholipids 1:14 *w*/*w* (total lipids); and 49% (*w*/*w*) lunasin extracted from soybean and added to the total lipids. Liposome characterization, as well as amaranth unsaponifiable matter and lunasin extraction, were described in our previous study [15]. The liposome formulation was adjusted every week after mice weighing.

For confocal microscopy, rhodamine-labeled liposomes were prepared using 1,2-dioleoyl-sn-glycero-3-phosphoethanolamine-*N*-(lissamine rhodamine B sulfonyl) (DOPE-Rhod): total lipids 1:43 *w*/*w* ratio. Different liposome preparations were used for cell treatment: amaranth unsaponifiable matter-loaded liposomes (UMLip), lunasin-loaded liposomes (LunLip), and lunasin and amaranth unsaponifiable matter-loaded liposomes (UM + LunLip), with the previously described ratios (DOPE-Rhod:total lipids 1:43 *w*/*w*).

#### 2.2.2. Confocal Experiments in B16-F10 Melanoma Cells to Elucidate the Endocytic Pathway

To determine the liposomes cell uptake, B16-F10 melanoma cells were grown in controlled temperature (37 °C) and atmosphere (5% CO_2_, 95% air) conditions. Endocytic pathway uptake was evaluated according to the protocol described by Kang et al. [22] with some modifications. Cells were seeded and incubated overnight in an Ibidi^TM^ µ-Dish 35 mm with a 1 × 10^5^ cells/mL confluence and treated with rhodamine-labeled liposomes loaded with UMLip, LunLip, and UM + LunLip. All lunasin loaded rhodamine-labeled liposomes were used following the IC_25_ value (0.62 mg/mL) based on our previous study [15].

Cells were pre-incubated for 60 min with 10 µg/mL chlorpromazine hydrochloride (clathrin-mediated endocytosis, CME, inhibition), or 54 µg/mL genistein (caveolae-mediated endocytosis, CvME inhibition), or 10 µg/mL cytochalasin D (macropinocytosis inhibition) [22]. After cell pre-incubation, liposomes solutions were added and incubated for 60 min. Afterwards, cells were washed two times and replaced with fresh medium. For nucleus staining, Hoechst 33342 was used following the manufacturer’s instructions and incubated for 30 min.

The cells were imaged using an LSM 880 confocal microscope (Carl Zeiss, Oberkochen, Germany), and captured with a 63×/1.4 Oil DIC objective. Images were scanned in high-resolution format (1912 × 1912 pixels). For rhodamine, the laser was set to 561 nm wavelength excitation and 583 nm wavelength emission. For Hoechst 33342, the laser was set to 350 nm wavelength excitation, and 461 nm wavelength emission, laser power of 0.23%. The fluorescence intensity was measured using Zen Black software (Carl Zeiss, Oberkochen, Germany). Single cells were measured by hand drawing the shape over the cell membrane aiming to avoid any signal from the background (Supplemental Figure S1).

Since the labeling was done for the cell nuclei and the liposomes aiming to avoid any other signal, those images with low liposome uptake were dark. Therefore, only for visual purposes, a gamma correction (1.5–3 with auto-adjust at 0.5 on white and 0 on black) for all the images accordingly to Song et al. [23] to visualize the dim objects without oversaturating the brightest objects.

#### 2.2.3. Animals for In Vivo Experiment and Experimental Design

The animal procedures and handling were conducted in accordance with the guidelines of the Institutional Animal Care and Use Committee (IACUC) at the University of Illinois at Urbana-Champaign, IACUC project number 21098.

Males have a higher risk to develop melanoma skin cancer [3]; it was also reported that melanoma tumor growth in female mice is lower than in male mice [24]. Therefore, fifty-eight male C57BL/6 mice aged 4–5 weeks were purchased from Envigo (Indianapolis, IN, USA), and grouped in 4 mice per cage. Animals had access to water and soybean flour-free food (Teklad Global 2016, 16% protein diet with 22% calories from protein, 12% calories from fat, and 66% calories from carbohydrates, Harlan Teklad, Madison, WI, USA) ad libitum. Mice were maintained in a 12:12 h light/dark cycle and treated humanely.

**Figure 1:**
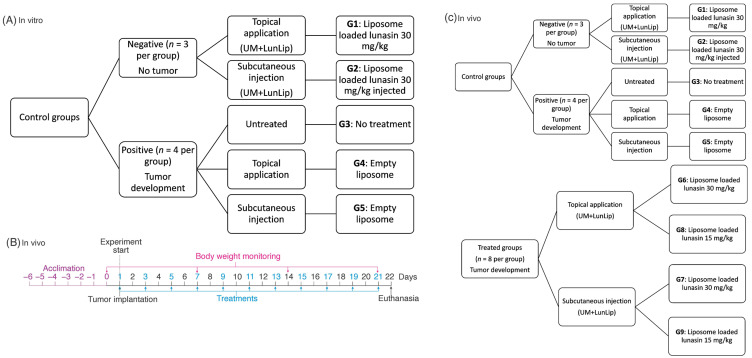
Experimental design. (**A**) Summary of the confocal in vitro experiment. (**B**) In vivo experimental timeline. (**C**) Groups and treatments in the in vivo study. UM + LunLip; liposomes loaded with soybean lunasin and amaranth unsaponifiable matter. DOPE-Rhod, 1,2-dioleoyl-sn-glycero-3-phosphocholine; DOPE-Rhod, 1,2-dioleoyl-sn-glycero-3-phosphoethanolamine-*N*-(lissamine rhodamine B sulfonyl). Negative control groups (non-tumor transplantation): G1 (liposomes loaded with 30 mg/kg BW lunasin topically applied), and G2 (liposomes loaded with 30 mg/kg BW lunasin subcutaneously injected); positive controls (with tumor transplantation): G3 (untreated positive group), G4 (empty liposomes topically applied), and G5 (empty liposomes subcutaneously injected); treated groups (with tumor transplantation): G6 (liposomes loaded with 30 mg/kg BW lunasin topically applied), G7 (liposomes loaded with 30 mg/kg BW lunasin subcutaneously injected), G8 (liposomes loaded with 15 mg/kg BW lunasin topically applied), and G9 (liposomes loaded with 15 mg/kg BW lunasin subcutaneously injected). At the end of the experiment (day 22) all mice survived.

To investigate the tumor development prevention, mice were randomly distributed into 9 groups (Figure 1). After 1-week acclimation, groups G1 and G2 were considered negative controls (healthy, no melanoma cells applied). Tumors were induced by subcutaneous injection in the right rear flank with 1 × 10^5^ B16-F10 melanoma cells in 100 µL PBS in groups G3–G9. Since the objective was to assess the tumor prevention, right after cell transplantation, treatments were started accordingly to Figure 1.

Treatments consisted of liposomes with 30 (G6 and G7) or 15 (G8 and G9) mg lunasin/kg BW via either topical (G6 and G8) or subcutaneous (G7 and G9) every other day. The controls G4 and G5 were treated with empty liposomes (only phospholipids) applied topically and subcutaneously every other day, respectively. The experiment duration after cell transplantation was 22 days. Some animals were euthanized on day 21 due to endpoint criteria. Euthanasia was performed with carbon dioxide asphyxiation followed by cervical dislocation and tumors were collected. All in non-tumor-bearing control groups mice were euthanized and analyzed, no malignancies were noticed during euthanasia nor IHC analysis in non-tumor-bearing control groups. Free lunasin topically applied was tested but there was no significance with regard to G3 (data no shown).

Mice weight was measured once per week. Tumor measurement was performed twice per week after starting on day 11th. Tumor size was measured using a digital caliper, and the volume was calculated using the formula [25]:

(1) $$Tumor\;volume=\;\frac{\left(A\times B^{2}\right)}{2}$$

where *A* = the largest value and *B* = the smaller value of both dimensions. Right before euthanasia, the presence of tumors was registered, tumors were measured, and after euthanasia tumors were excised, weighed, and fixed in 10% neutral buffered formaldehyde for 48 h.

Samples were processed through graded alcohols and xylene into paraffin using a Sakura Tissue-Tek VIP 6 tissue processor (Sakura Finetek Inc., Torrence, CA, USA). They were then embedded using a Sakura Tissue-Tek TEC 5 Embedding System (Sakura Finetek Inc., Torrence, CA, USA). Five-micron sections were then cut from the paraffin blocks with a Leica RM2125 RTS Microtome (Leica Biosystems, Buffalo Grove, IL, USA) and were mounted on glass slides.

For all the stains, the slides were deparaffinized with xylene and rehydrated through an ethyl alcohol series.

#### 2.2.4. Histochemical and Immunohistochemical Assays

Aiming to know the cell proliferation (Ki-67), apoptosis (caspase-3) and GSK-3β expression, immunohistochemistry (IHC) was performed.

For hematoxylin and eosin (H&E), slides were placed in modified Harris hematoxylin for 2 min, differentiated in acidified 70% ethanol (0.5% HCl), and blued in 0.5% ammonia water. Water rinse was followed by 95% ethanol for 30 s in eosin The slides were dehydrated, cleared, and mounted.

For GSK-3β, slides were pretreated in 1 mM ethylenediaminetetraacetic acid pH 9.0 was performed using a pressure cooker. Blocked for endogenous peroxidase using 3% hydrogen peroxide for 10 min followed by protein blocking using rodent block M. The sections were incubated with GSK-3β antibody. After rinsing in tris-buffered saline, the secondary antibody (mouse-on-mouse HRP-Polymer) was applied for 30 min. This was followed by a 3,3′-diaminobenzidine (DAB) chromogen and then counterstained with hematoxylin, dehydrated, cleared, and mounted.

For caspase-3, slides were pretreated in 1 mM ethylenediaminetetraacetic acid pH 9.0 was performed using a pressure cooker. Blocked for endogenous peroxidase using 3% hydrogen peroxide for 10 min followed by protein blocking using background sniper. The sections were incubated with a caspase-3 antibody. After rinsing in tris-buffered saline, the secondary antibody (rabbit-on-rodent HRP Polymer, Biocare Medical, Pacheco, CA, USA) was applied for 30 min. This was followed by DAB chromogen and then counterstained with hematoxylin, dehydrated, cleared, and mounted.

For Ki-67, slides were pretreated in 1 mM citrate pH 6.0 was performed using a pressure cooker. Blocked for endogenous peroxidase using 3% hydrogen peroxide for 10 min followed by protein blocking using background sniper. The sections were incubated with a Ki-67 antibody. After rinsing in tris-buffered saline, the secondary antibody (rabbit-on-rodent HRP Polymer) was applied for 30 min. This was followed by DAB chromogen and then counterstained with hematoxylin, dehydrated, cleared, and mounted.

Slides with tissue sections were deparaffined with xylene, stained, and imaged in a NanoZoomer Digital Pathology System C96000-12 (Hamamatsu, Hamamatsu, Japan). The images were visualized and obtained using the NDP.view v2.9.25 Viewing software (Hamamatsu, Hamamatsu, Japan).

IHC images were analyzed at 20× using the Fiji app (NIH, Bethesda, MD, USA) using threshold and normalizing the data per cell nuclei [26] with slight modifications. After applying color deconvolution using the “H DAB” option, colors 1 (blue for hematoxylin) and 2 (brown for DAB), a threshold was applied for both colors. For color 2, the optimum threshold value per image was averaged and all images were analyzed using the average for comparison purposes. However, for color 1 every threshold was independently set to include as many nuclei as possible avoiding background noise that could interfere.

For histochemical analyses, slides were stained with hematoxylin and eosin. Images were analyzed for necrosis using the artificial neural network pixel classifier [27] from the open-source QuPath v0.3.0 software [28]. Tumor cells were selected in red color, necrotic tissue in black, and stroma in green (Supplemental Figure S2).

#### 2.2.5. Statistical Analysis

ROUT test (Q = 1%) was performed on the data sets and detected 2 outliers, G7 mouse 3 and G9 mouse 3 which were taken out from all the analysis. Outliers test was performed using GraphPad Prism 8 (GraphPad Software Inc., San Diego, CA, USA), as well as Kruskal–Wallis tests, and one-way ANOVA, multiple comparisons were tested with Dunn and Tukey post hoc tests, respectively. Experimental data were tested for normality and analyzed either by using ANOVA or Kruskal–Wallis. A *p*-value < 0.5 was considered statistically significant. Results are represented as the mean ± S.E.M.

## 3. Results

### 3.1. Liposome Uptake in Melanoma Cells B16-F10

To determine the liposome uptake pathway used by B16-F10 melanoma cells, three endocytic pathways were inhibited (Figure 1). Clathrin-mediated endocytosis (CME) was inhibited by chlorpromazine hydrochloride, a cationic amphiphilic molecule that inhibits adaptor protein 2 (AP2). AP2 is recruited to the cell surface binding to phosphatidylinositol 4,5-bisphosphate, leading to conformational changes that expose the cargo binding sites. AP2 binds to clathrin triskelion to allow endocytosis [29,30,31].

Caveolae-mediated endocytosis (CvME) was inhibited by genistein, a tyrosine kinase inhibitor that prevents cytoskeletal actin polymerization and caveola vesicle internalization. Genistein prevented dynamin II recruitment. The role of dynamin II is in the vesicle scission of the membrane [31].

Macropinocytosis was inhibited by cytochalasin D, a mycotoxin that prevents actin polymerization by binding to the F-actin barbed end [30,32].

**Figure 2:**
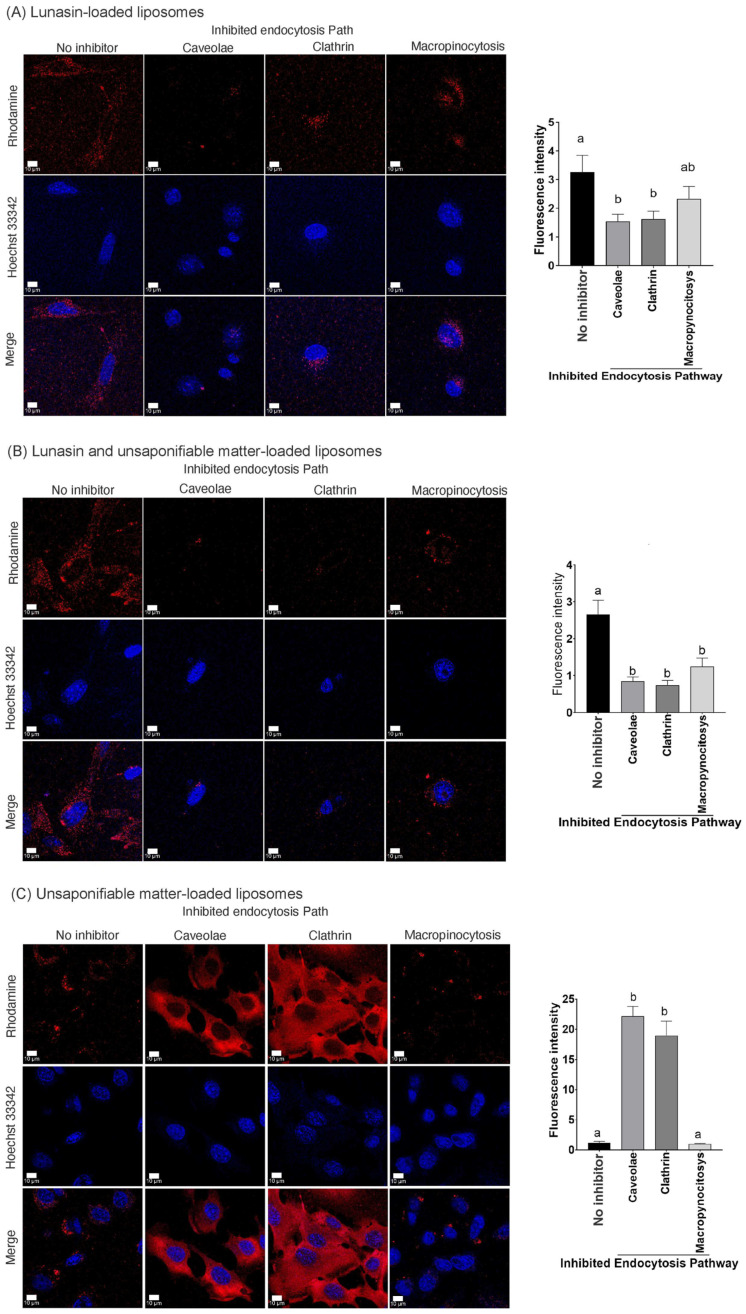
Preparation of different liposomes absorbed in B16-F10 melanoma cells after the inhibition of clathrin-mediated endocytosis (CME), caveolae-mediated endocytosis (CvME), and macropinocytosis. (**A**) Micrographs and intensity chart of liposomes loaded with lunasin. (**B**) Micrographs and intensity chart of liposomes loaded with lunasin and unsaponifiable matter. (**C**) Micrographs and intensity chart of liposomes loaded with unsaponifiable matter. The rhodamine fluorescence (red channel) is proportional to liposome uptake. The higher the uptake, the brightest and more defined the cell shape as seen in section (**C**). The intensity analysis was performed manually considering the fluorescence inside the cells and aiming to avoid the signal quantification of liposomes outside the cells. Only for visual purposes and owing to the dark background generated by the low uptake of liposomes in 60 min, a gamma enhancement (**A**,**B**) gamma = 3, and (**C**) gamma = 1.5 with auto adjust in white 0.5 and black 0 was applied. The scale bars in the image was 10 μm. Statistical analysis was performed using ANOVA with Tukey’s post hoc test for multiple comparisons. In the graphs, different letters indicate statistically significant differences. The data are expressed as the mean ± standard error. Statistical significance was set at *p* < 0.05. CME was inhibited by chlorpromazine hydrochloride, a cationic amphiphilic molecule that inhibits adaptor protein 2. Genistein inhibited CvME. Macropinocytosis was inhibited by cytochalasin D, a mycotoxin that prevents actin polymerization by binding to the F-actin barbed end. No endocytosis inhibitor was added to control cells.

The liposome loaded with lunasin (LunLip) (Figure 2A) and liposomes loaded with soybean lunasin and amaranth unsaponifiable matter (UM + LunLip) (Figure 2B) treatments decreased liposome uptake (*p* < 0.05) compared to the non-inhibited controls. However, there was no difference in liposome uptake among the three inhibited endocytosis pathways (CME, CvME, and macropinocytosis) in either the LunLip or UM + LunLip treatments. Liposome uptake decreased while inhibiting CME and CvME at almost the same intensity in both the LunLip (CME, 50%; CvME, 47%) (Figure 2A) and UM + LunLip (CME, 20%; CvME, 32%) treatments (Figure 2B). In LunLip, there was no difference from the no inhibitor (positive) control, while macropinocytosis was inhibited. This suggests that liposomes loaded with lunasin were uptake into the cell via the CME or CvME pathways. CME followed by CvME is the preferred route for nanoparticle uptake [33].

Rhodamine was bound to the liposome bilayer; therefore, the red signal shown in Figure 2 belongs to liposomes. However, owing to the low uptake of liposomes after 1 h of treatment in cells B16-F10, a gamma contrast of 1.5–3 was used only for visual purposes, not for intensity analysis [23]. An intensity analysis was performed for each individual cell, as shown in Supplemental Figure S1.

In contrast to the previously described results (Figure 2A,B), liposomes loaded with unsaponifiable matter (UMLip) (Figure 2C) exhibited a significant increase (*p* < 0.05) in uptake compared to the no-inhibitor control. The inclusion of squalene in nanoparticles improved their uptake through the macropinocytosis pathway [34]. Trung et al. [35] showed a similar uptake behavior of nanoparticles prepared with gemcitabine and squalene in HeLa cells incubated at 4 °C. The authors reported that compared with the control, uptake increased while inhibiting CME and CvME.

Abrunhosa et al. [36] reported strong electrostatic surface interactions between two peptides and the liposome surface. Therefore, a possible explanation for the increase in the relative fluorescence units in UMLip (Figure 2C) compared with LunLip and UM + LunLip (Figure 2A,B) is that negatively charged amino acids (aspartic acid tail) in lunasin can interact with DOPG, leading to lunasin-liposome surface interaction. This interaction could form lunasin-coated liposomes. However, further investigation is needed to clarify whether lunasin interacts with liposome surfaces.

### 3.2. Tumor Development in an Allograft Model Using C57BL/6 Mice Subjected to Different Liposome Treatments

**Figure 3:**
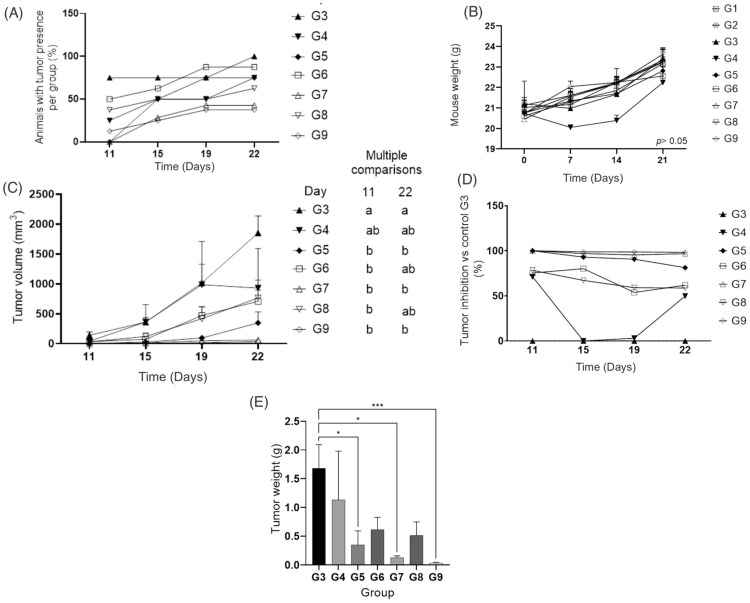
Mouse model tumor development and body weight. (**A**) Tumor presence per group by measurement day. (**B**) Mouse body weight throughout the experiment; significant differences were shown only on day 21. (**C**) Tumor volume throughout the experiment; different letters show significant differences among the groups per measurement day. (**D**) Tumor inhibition with respect to the positive control group (G3). (**E**) Tumor weight by group. Different letters indicate statistical differences among the groups. The data were analyzed using the Kruskal–Wallis test, and Dunn’s test was used for multiple comparisons. The data are expressed as the mean ± standard error; *p* < 0.05 was considered statistically significant. *, *p* < 0.05; ***, *p* < 0.001. In the multiple comparisons section, different letters indicate statistically significant differences. Negative control groups (non-tumor transplantation): G1 (liposomes loaded with 30 mg/kg BW lunasin topically applied), and G2 (liposomes loaded with 30 mg/kg BW lunasin subcutaneously injected); positive controls (with tumor transplantation): G3 (untreated positive group), G4 (empty liposomes topically applied), and G5 (empty liposomes subcutaneously injected); treated groups (with tumor transplantation): G6 (liposomes loaded with 30 mg/kg BW lunasin topically applied), G7 (liposomes loaded with 30 mg/kg BW lunasin subcutaneously injected), G8 (liposomes loaded with 15 mg/kg BW lunasin topically applied), and G9 (liposomes loaded with 15 mg/kg BW lunasin subcutaneously injected). At the end of the experiment (day 22) all mice survived.

B16 melanoma model is the most used model for in vivo experiments [37]. After B16-F10 melanoma cell transplantation, tumor development was uneven among the treated groups, depending on the liposome composition and administration method. By experimental day 11, only two of the eight transplanted groups had not developed tumors (G5 and G7). However, by the end of the experiment (day 22), at least one mouse in each group had developed at least one tumor (Figure 3A). All mice in G3 (tumor-bearing, no treatment) developed tumors by the end of the experiment.

Lunasin solution (unencapsulated) topically applied was additionally tested. There was no difference comparing with the G3 (data not shown) which indicates the lack of penetration of lunasin through the skin as a free peptide. The greatest challenge for lunasin delivery through transdermal application is to penetrate the epidermal stratum corneum because it functions as a barrier. However, when lunasin was loaded into the liposomes, the penetration improved owing to the compatibility of the phospholipids of the liposomes with the cells [38].

Over time (day 21), there was no statistical difference in mouse body weight (BW) among groups (Figure 3B).

Groups treated with liposomes (Figure 3C) (G4–G9) after day 22 showed a smaller tumor volume (from 32 mm^3^ to 707 mm^3^) than the tumor-bearing untreated group (G3, tumor volume 1852 mm^3^).

On day 11, the tumor volume in G3 was 139 mm^3^. However, the tumor volume of G9 was unmeasurable because the tumors were too small for the caliper. Meanwhile, on day 22, measurable tumors in G9 had a volume of 111.69 mm^3^, in contrast to G3, which developed an average tumor volume of 1851.58 mm^3^. It is important to highlight that some tumors were not measurable with the caliper owing to their small size.

Tumor inhibition in all groups was related to the tumor volume of the control G3 (Figure 3D). On the first day tumors were measured (day 11), all animals in G3 developed tumors, but only one in G4; the mean tumor volume was 139.5 mm^3^ for G3 and 40.13 mm^3^ for G4 (71% less tumor volume in comparison to G3). On day 15, the tumor development of G4 accelerated. The tumor volume was 360.62 mm^3^ for G3 and 362.11 mm^3^ for G4, which gave 0% tumor inhibition for G4. On day 19, the tumor volume was 1015.61 mm^3^ for G3 and 928.85 mm^3^ for G4, which means a 3% inhibition for G4. The tumor development in G3 was augmented by day 22, reaching a volume of 1851.58 mm^3^. Meanwhile, tumor development in the G4 plateau (929 mm^3^) translated to a 49% tumor inhibition in G4.

Groups G7 and G9, which were injected subcutaneously exhibited a significant inhibition (*p* < 0.05) in tumor volume (Figure 3D) of up to 99% with regard to the positive control (tumor-bearing untreated group, G3). In an in vivo melanoma study using B16-F10 melanoma cells as tumor initiators, the intraperitoneal injection of lunasin (30 mg/kg BW) decreased tumor volume by 55% by day 22 [14]. In other melanoma mouse model studies [39,40], the largest tumor volumes achieved were approximately 100 mm^3^ and 1000 mm^3^, respectively, which are comparable to the tumor volumes developed in this study.

In liposomes loaded with lunasin (15 mg/kg BW) and amaranth unsaponifiable matter (G9), the tumor volume was inhibited by 99% with respect to G3. Devapatla et al. [14], reported that lunasin administered via intraperitoneal injection with a 10 mg/kg BW did not result in a significant reduction in the tumor volume compared with the control in tumors developed with lung carcinoma cells LLC.

Even though, the empty liposomes (phospholipids without any other component) did not show cytotoxicity in B16-F10 cells in our previous study [15], empty liposomes in the animal model worked well either topically or subcutaneously applied, showing a smaller tumor volume (G4 = 928.85 mm^3^ and G5 = 346 mm^3^) than those in the untreated group G3 (Figure 3C). König et al. [41] reported anticancer activity in an animal model of basal cell carcinoma skin cancer when empty liposomes were applied, non-statistically significance were shown between the empty liposomes and the synthetic bacterial lipoprotein and TLR1/2 agonist Pam3CSK4 (BLP)-loaded liposomes. The anticancer effect was mediated particularly because empty liposomes increased the presence of tumor-associated macrophages. Additionally, empty liposomes prepared with phosphatidylcholine showed antimetastatic activity due to the alteration of fatty acid profile in tumor environment, this alteration could affect the generation of lipid second messengers which are crucial for metastasis [42]. Even tough group G5 (black rhombus in Figure 3) prevented the tumor development (81%), it was not better than the liposomes loaded with amaranth unsaponifiable matter and soybean lunasin (99%).

Groups G7 and G9 behaved similarly to the control groups G1 and G2 (no tumors) in terms of tumor inhibition (Figure 3D). These results showed that liposomes loaded with lunasin and unsaponifiable matter were effective against the appearance of tumors meaning that UM + LunLip effectively prevented melanoma tumor development.

To test the effectiveness of the liposomes through two administration approaches, an invasive and non-invasive subcutaneous injection and topical application, respectively, were used. By the end of the experiment, topically applied liposomes (G8) and subcutaneously applied liposomes (G9) achieved inhibition rates of up to 59% and 99%, respectively (Figure 3D). Jose et al. [43] showed that liposomes applied via intra-tumoral injection and iontophoresis (which aims to increase the drug skin penetration applying a low electric current through the skin using two electrodes [44]) were more effective in lowering the tumor volume than the topical application owing to permeation.

Topical application depends on dermal absorption and allows a compound to permeate through the skin layers. Nanoparticles penetrate the skin through the epidermal membrane, capillary follicles, sebaceous glands, sweat glands, and intracellular hydrophobic channels [45]. As skin thickness varies among different species, topical treatments should be validated in each species. In mice, the skin thickness ranges from 211 μm to 671 μm [46]. The groups treated with the topical application of UM + LunLip were G4, G6, and G8 (Figure 3C). Even though the subcutaneous application was better than topical application, tumor prevention was higher than 50% for those animals treated with liposomes applied topically. Jose et al. [43] showed that after 4 h of contact with pig ear skin, liposomes prepared with curcumin reached a penetration depth of 50 μm.

**Figure 4:**
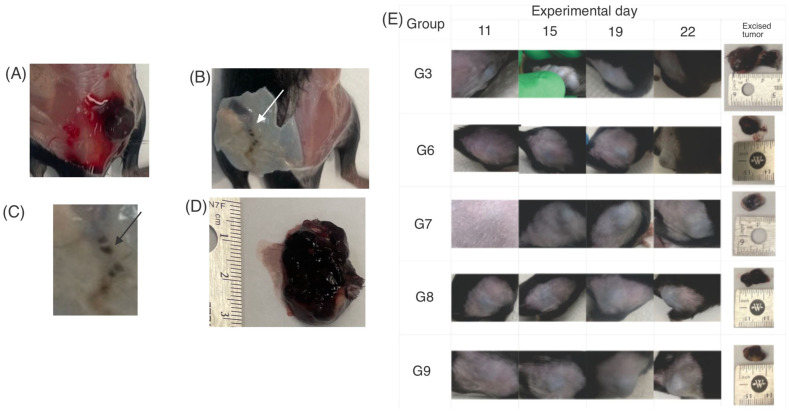
Tumor development and presence by groups. (**A**) Tumor with irrigation at experimental day 22 (G7). (**B**) Growing tumor without irrigation at experimental day 22 (G9, white arrow). (**C**) The arrow points to the tumor equivalent to carcinoma in situ (G9). (**D**) Invasive tumor (G4). (**E**) Tumor development, comparison among the untreated positive group (G3), and groups treated with liposomes loaded with lunasin 30 mg/kg body weight administered topically (G6), subcutaneously injected (G7), as well as liposomes loaded with lunasin 15 mg/kg body weight administered topically (G8) and subcutaneously injected (G9).

In terms of tumor weight (Figure 3E), there was a difference (*p* < 0.05) between the untreated group (G3) and the groups treated with liposomes subcutaneously applied (G5, G7, and G9). There was no significant difference between the treatments with different lunasin concentrations. The tumor weight was affected by empty liposomes as well, as previously stated, this behavior can be explained because empty liposomes increased the presence of tumor-associated macrophages [42]. It is important to highlight that during euthanasia, the animals with larger tumor volumes (G3) showed irrigation of the tumor (Figure 4A) compared with those with melanocytic lesions (G9) (Figure 4B). In some mice, the skin area (visual observation) could influence the tumor weight and contributed more than the melanocytic lesions (Figure 4C), compared with the developed tumors (Figure 4D).

Some tumors could be considered melanoma in situ (Figure 4B) instead of invasive tumors (Figure 4D). Carcinoma in situ has been described as a tumor between 0.5 mm and 1 mm in diameter [47].

Figure 4E shows the tumor size and development in groups G3, and G6–G9. The group that developed the largest tumor was G3, compared to those treated with liposomes loaded with lunasin and unsaponifiable matter (G6–G9); by day 22, there was a tumor inhibition of 62% (G6) and 59% (G8) with topical application, as well as 97% (G7) and 99% (G9) with subcutaneous application in comparison to G3.

During euthanasia, it was observed that some mice, especially those in groups G7 and G9, did not develop tumors. Some mice at G6, G7, and G9 did not show irrigation (Figure 4B,C). Therefore, an angiogenesis marker should be measured in the future to determine whether angiogenesis is affected. Angiogenesis is important for tumor growth and metastasis [48].

Liposomes can improve drug delivery to cells, leading to a reduction in drug dosage [49]. Therefore, UM + LunLip did not show any difference in tumor inhibition when applied at either 15 or 30 mg lunasin/kg BW. The cytotoxicity of lunasin in melanoma cells B16–F10 increased when loaded into liposomes [15]. To determine the inhibition of tumor development, three markers were tested: Ki-67, caspase-3, and GSK-3β.

#### 3.2.1. Immunohistochemistry (IHC) Image Analysis

**Figure 5:**
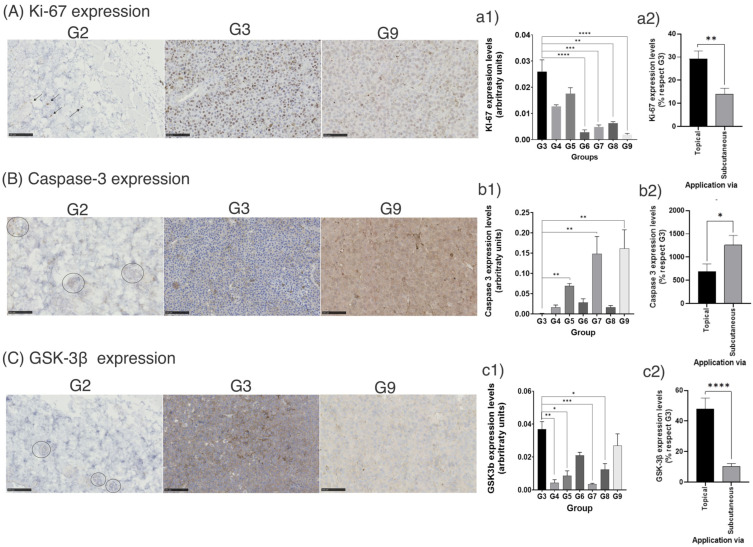
Immunohistochemistry expression of Ki-67, caspase-3, and GSK-3β. (**A**) Ki-67 expression micrographs. (**a1**) Ki-67 expression level per group. (**a2**) Ki-67 expression level per application method. (**B**) Caspase-3 expression micrographs. (**b1**) Caspase-3 expression level per group. (**b2**) Caspase-3 expression level per application method. (**C**) GSK-3β expression micrographs. (**c1**) GSK-3β expression level per group. (**c2**) GSK-3β expression levels per application method. Micrographs show the expression in the treated group G9 and controls (G2 and G3). Bars show the expression levels of the different markers in the tumor- bearing groups (G3–G9). In the graphs, *, *p* < 0.05; **, *p* < 0.01; ***, *p* < 0.001; ****, *p* < 0.0001. The data are expressed as mean ± standard error; control G2 (no tumor) treated with liposomes loaded with 30 mg/kg BW lunasin and unsaponifiable matter applied subcutaneously; G3, untreated positive group (tumor-bearing mice); G9, liposomes loaded with 15 mg/kg BW lunasin subcutaneously injected. The scale line in the image is 100 μm in length. Circles or arrows in micrographs indicate positive areas for each marker.

Images obtained with IHC showed that lunasin-loaded liposomes inhibited Ki-67 expression (*p* < 0.001) compared with the untreated control (G3) (Figure 5A). The empty liposomes (G5) did not yield significantly different results compared with the control (G3, *p* > 0.05). The findings suggest that liposomes loaded with lunasin and unsaponifiable matter (G6–G9) inhibited the tumor cell cycle progression. Ki-67 is a proliferation marker that is expressed in all cell cycle phases (G1, S, M, and G2), but not in G0; it can identify all cycling cells [50]. Increased Ki-67 has been correlated with recurrence and metastasis, especially in thick tumors [51].

There were no major differences when the skin from non-bearing tumor controls (G1 and G2) was observed for IHC marker evaluation (Figure 5A–C in G1 and G2).

Most drugs used to treat cancer aim to induce apoptosis [52]. The arginine-glycine-aspartic acid (RGD) motif in peptides induces the activation of caspase-3. Specifically, lunasin, which has an RGD domain within its peptide sequence, has been reported to activate caspase-3 in L1210 leukemia cells [53]. In this study, caspase-3 overexpression was observed when comparing the tumor-bearing untreated control (G3) with G7 and G9 (tumor-bearing group treated with liposomes subcutaneously administered with 30 mg and 15 mg of lunasin/kg BW, respectively) (*p* < 0.05) (Figure 5B).

In the caspase family, caspase-3 is recognized as an important marker of apoptosis [54]. In the intrinsic apoptosis pathway, Bax is inserted into the mitochondrial membrane forming channels, where the cytochrome-c molecules escape from the inside of the mitochondria, leading to the formation of apoptosomes. During this process, procaspase-9 is activated. Caspase 9 cleaves procaspase-3, producing the proteolytic degradation of its target substrate, leading to cell apoptosis [55].

However, caspase-3 overexpression in melanoma development is still controversial as it has been reported that apoptotic cells potentiate viable cell proliferation [56] which is similar to apoptosis-induced proliferation. It is important to highlight that apoptosis is not a necrotic effect, apoptosis is the controlled death of the cell without spillage of the cell content forming apoptotic bodies instead. Necrosis is the uncontrolled cell death induced by external factors (such as hypoxia) producing the cell membrane rupture causing the spillage of the cell content [57].

The UM + LunLip treatments inhibited GSK-3β expression (Figure 5C), as suggested in our previous study [16]. The subcutaneous application of liposomes at 30 mg lunasin/kg BW (G7) significantly inhibited GSK-3β expression (*p* < 0.001). GSK-3β is involved in cell differentiation, cell cycle progression, apoptosis, growth metabolism, and the modulation of transcription factors that regulate cell survival or death, among others. Depending on the cell type and signaling environment, this protein can function as a tumor promoter or tumor suppressor [58]. When GSK-3β is inhibited, it has been reported that the mitochondria-initiated apoptosis pathway is deactivated blocking the cytochrome c release and caspase-3 activation [59]. However, it has been suggested that GSK-3β inhibition induces β-catenin expression [60,61], leading to apoptosis [60].

As shown in Figure 5a2,b2,c2, the groups treated with UM + LunLip subcutaneously applied (G7 and G9) had the best response related to the three different markers. Caspase-3 expression was 17.87-fold higher than tumors in G3 when UM + LunLip was applied subcutaneously (G7 and G9) and 6.9-fold higher than tumors in G3 when applied topically (G6 and G8) (*p* < 0.05). The subcutaneous application (G7 and G9) of liposomes resulted in a 20% under-expression of GSK-3β and Ki-67 with respect to G3. Meanwhile, the topical application (G6 and G8) of UM + LunLip resulted in a 48% and 29% under expression of GSK-3β (*p* < 0.0001) and Ki-67 (*p* < 0.01), respectively.

#### 3.2.2. H&E Image Analysis Resulted in Large Necrotic Tumor Areas

Necrosis is a marker of aggressive tumors (larger tumor diameter and metastasis). Necrosis has been suggested to be a strong and independent predictor of poor survival. Necrosis is strongly associated with aggressive tumor features such as ulceration, vascular invasion, and reduced patient survival [62]. Therefore, similar to other cancer types, necrosis measurement is recommended for cutaneous melanoma [63].

**Figure 6:**
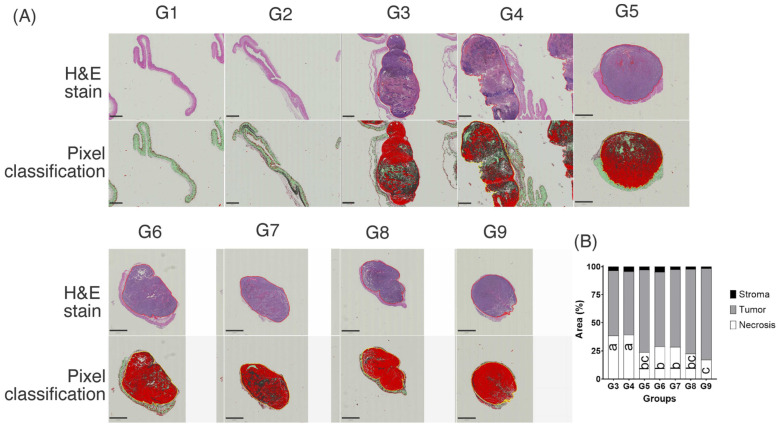
Hematoxylin and eosin (H&E) staining. (**A**) H&E staining of some tumors as examples of the different groups and the pixel classification; red-colored pixels show the tumor viable cells, black-colored pixels show the necrotic cells, and green-colored pixels show the stroma. (**B**) Bars show the average percentage of necrotic cells, viable cells, and stroma per individual tumor per group (G3–G9). Images were taken from QuPath software set at a 0.5× magnification; the scale bar is equal to 2 mm. An ANOVA test was computed to compare the necrotic area percentage between groups. In the graph, different letters inside the bars indicate differences (*p* < 0.0001) in the necrotic area between the groups. Controls G1 and G2 (no tumor developed) treated with liposomes loaded with lunasin 30 mg/kg BW and unsaponifiable matter, either applied topically or injected subcutaneously, respectively; G3, untreated positive group (tumor-bearing mice); G4, empty liposomes topically applied (tumor-bearing mice); G5, empty liposomes injected subcutaneously (tumor-bearing mice); treated groups (with tumor transplantation): G6, liposomes loaded with 30 mg/kg BW lunasin applied topically applied; G7, liposomes loaded with 30 mg/kg BW lunasin injected subcutaneously; G8, liposomes loaded with 15 mg/kg BW lunasin applied topically; G9, liposomes loaded with 15 mg/kg BW lunasin injected subcutaneously. Pearson’s correlation showed a negative correlation (*r* = −0.83, *p* < 0.05) between tumor size and caspase-3 expression. Therefore, the caspase-3 expression was higher in tumors with a smaller size and lower in tumors with a higher tumor volume. Tumors treated with UM + LunLip under-expressed the proliferation markers Ki-67 and GSK-3β and overexpressed caspase-3 (Figure 7). In general, these results suggest the activation of the apoptosis pathway through caspase-3 overexpression, which could be initiated after GSK-3β inhibition. The UM + LunLip treatments affected cell cycle progression, as indicated by the under-expression of Ki-67.

Figure 6a shows the necrotic area of a representative tumor per group and the pixel classifier using QuPath (Supplemental Figure S2). The presence of necrosis is an important marker for the estimation of tumor aggressiveness. Our results showed that the larger the tumor, the higher the number of necrotic cells (Figure 6). After measuring the necrotic area (%) in relation to the total individual tumor (Supplemental Figure S2), it was shown that tumors in G3 had a higher percentage of necrotic cells. In contrast, G9, the group with the smallest tumor volume, had a lower percentage of necrotic cells per tumor (*p* < 0.0001). Therefore, UM + LunLip diminished the necrotic area in tumors compared with that in the tumor-bearing group G3 (Figure 6B). With regard to lunasin concentration, UM + LunLip subcutaneously administered at 15 mg/kg BW (G9) decreased (*p* < 0.0001) the necrotic area with respect to UM + LunLip loaded with 30 mg/kg BW (G8).

Tumor necrosis is related to hypoxia and nutrient deficiency, which promote angiogenesis and tumor progression; consequently, tumor necrosis is related to vascularization in tumors; necrotic areas have also been related to the growth speed of the tumors, with the fastest growth being the most inadequate vascularization [64,65]. Thus, a larger melanoma tumor size is related to a larger necrotic area within the tumor [66]. Hence, the observations in the present study support the relationship between tumor size and necrotic area; the groups with the largest tumor volume (Figure 3C) had the largest necrotic area (Figure 6).

The tumor-bearing untreated group G3 had a higher necrotic percentage with respect to the total tumor (38%) than the groups treated with UM + LunLip (17% for G9). Even though, 100% of the animals survived until the end of the experiment, the survival of animals with larger tumor volumes (G3) is more likely to be lower than those with lower tumor volume (G6–G9) since the larger necrotic area within the tumors indicates that those tumors are more aggressive.

## 4. Conclusions

The results of this study showed that UMLip was absorbed through macropinocytosis, whereas liposomes carrying lunasin (UM + LunLip) were absorbed through CME and CvME. The uptake of UMLip increased in the presence of squalene. However, further research is needed to confirm the interaction between lunasin and the liposome bilayer.

To our knowledge, this in vivo study is the first to demonstrate the ability of UM + LunLip to modulate markers involved in melanoma development. Tumor development was inhibited by up to 99% when UM + LunLip was applied subcutaneously compared to tumor-bearing untreated animals.

Caspase-3 was overexpressed in animals treated with UM + LunLip, and these groups presented the smallest tumor volume. The under-expression of Ki-67 was observed in the groups treated with UM + LunLip, suggesting cell cycle arrest. Animals with the smallest tumor volume and a smaller number of tumors exhibited the highest Ki-67 inhibition. Further research is needed to fully understand the effects of UM + LunLip in melanoma prevention.

Subcutaneous application was the best way to deliver UM + LunLip to tumor cells; it resulted in the smallest tumor volume, higher expression of Caspase-3, and lower expression of GSK-3β and Ki-67.

The findings approved our hypothesis: the soybean peptide lunasin and the amaranth unsaponifiable matter as a squalene source transported into liposomes effectively prevent melanoma tumor development in an in vivo model of C57BL/6 mice.

In future research, we intend to measure vascular and other markers to investigate angiogenesis development, as well as elucidate more in-deep the pathway that promotes tumor prevention, which was not among the objectives of the present study.

## Figures and Tables

**Figure 7:**
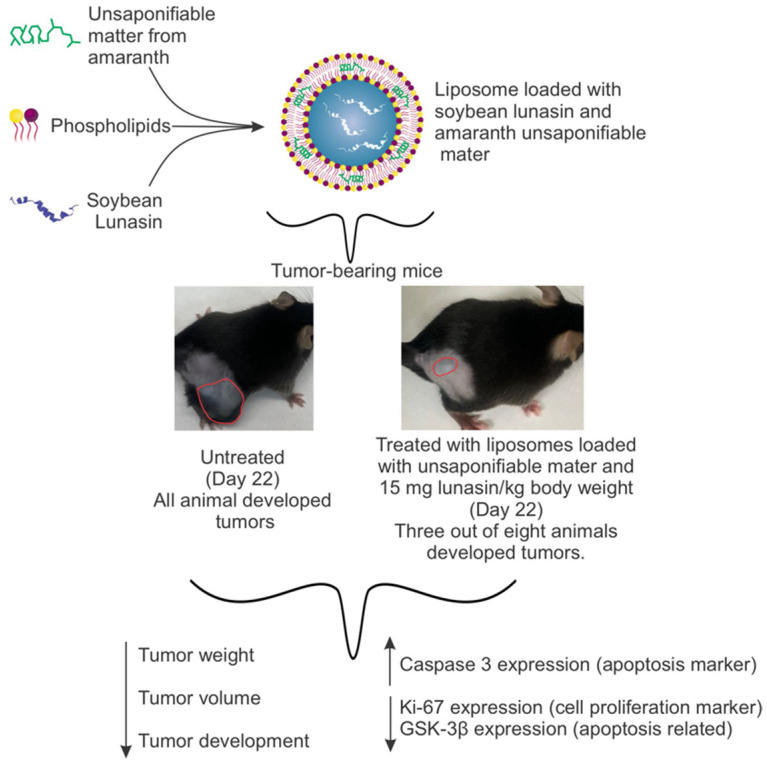
Outcomes after treatments using liposomes loaded with amaranth unsaponifiable matter and soybean lunasin applied to the melanoma in vivo model.

## Data Availability

Data is contained within the article or supplementary material.

## Supplementary Materials

The following are available online at https://www.mdpi.com/article/10.3390/pharmaceutics14102214/s1, Figure S1: Example of hand drawing cell membrane for confocal intensities analysis to avoid quantification of the liposomes located outside the cells using the Zeiss Zen Black software. Figure S2: QuPath analysis for necrotic determination. In magnification 0.39× can be shown the workflow for setting up the training pixel classifier. With the wand tool several areas for necrotic (black arrows and shadows), viable cells (red shadows), and stroma (green arrows and shadows) should be set. Once the pixel classification was trained properly, the tumor edges should be selected and the different pixels for necrosis, viable cells and stroma should be measured. In magnification 2.9× and 20× can be shown the pixel classification in better detail. Black arrows and shadows show necrotic areas, green arrows and shadows show stoma, and red shadows show viable cells.

## References

[1] Di Martile M., Garzoli S., Ragno R., Del Bufalo D. (2020). Essential oils and their main chemical components: The past 20 years of preclinical studies in melanoma. Cancers.

[2] Zhang X., Hu Z., Wang X., Li L., Zhu B., Lin X., Zhang J., Hua Z. (2021). ANXA10 promotes melanoma metastasis by suppressing E3 ligase TRIM41-directed PKD1 degradation. Cancer Lett..

[3] American Cancer Society Melanoma Skin Cancer Statistics. https://www.cancer.org/cancer/melanoma-skin-cancer/about/key-statistics.html.

[4] Cheng L., Lopez-Beltran A., Massari F., MacLennan G.T., Montironi R. (2018). Molecular testing for BRAF mutations to inform melanoma treatment decisions: A move toward precision medicine. Mod. Pathol..

[5] Oliveira R.D., Celeiro S.P., Barbosa-Matos C., Freitas A.S., Cardoso S.M., Viana-Pereira M., Almeida-Aguiar C., Baltazar F. (2022). Portuguese propolis antitumoral activity in melanoma involves ROS production and induction of apoptosis. Molecules.

[6] Rudd C.E., Chanthong K., Taylor A. (2020). Small Molecule Inhibition of GSK-3 Specifically Inhibits the Transcription of Inhibitory Co-receptor LAG-3 for Enhanced Anti-tumor Immunity. Cell Rep..

[7] Wu P.K., Hong S.K., Park J.I. (2021). Mortalin depletion induces MEK/ERK-dependent and ANT/CypD-mediated death in vemurafenib-resistant B-RafV600E melanoma cells. Cancer Lett..

[8] Domoto T., Uehara M., Bolidong D., Minamoto T. (2020). Glycogen synthase kinase 3β in cancer biology and treatment. Cells.

[9] Mirzavi F., Barati M., Soleimani A., Vakili-Ghartavol R., Jaafari M.R., Soukhtanloo M. (2021). A review on liposome-based therapeutic approaches against malignant melanoma. Int. J. Pharm..

[10] Castañeda-Reyes E.D., de Jesus Perea-Flores M., Davila-Ortiz G., Lee Y., de Mejia E.G. (2020). Development, characterization and use of liposomes as amphipathic transporters of bioactive compounds for melanoma treatment and reduction of skin inflammation: A review. Int. J. Nanomed..

[11] Li Y., Gao L., Tan X., Li F., Zhao M., Peng S. (2016). Lipid rafts-mediated endocytosis and physiology-based cell membrane traffic models of doxorubicin liposomes. Biochim. Biophys. Acta Biomembr..

[12] Karami N., Moghimipour E., Salimi A. (2018). Liposomes as a novel drug delivery system: Fundamental and pharmaceutical application. Asian J. Pharm..

[13] Shidal C., Al-Rayyan N., Yaddanapudi K., Davis K.R. (2016). Lunasin is a novel therapeutic agent for targeting melanoma cancer stem cells. Oncotarget.

[14] Devapatla B., Shidal C., Yaddanapudi K., Davis K.R. (2017). Validation of syngeneic mouse models of melanoma and non-small cell lung cancer for investigating the anticancer effects of the soy-derived peptide Lunasin. F1000Research.

[15] Castañeda-Reyes E.D., de Mejia E.G., Eller F.J., Berhow M.A., de Jesús Perea-Flores M., Dávila-Ortíz G. (2021). Liposomes loaded with unsaponifiable matter from Amaranthus hypochondriacus as a source of squalene and carrying soybean lunasin inhibited melanoma cells. Nanomaterials.

[16] Gonzalez De Mejia E., Castañeda-Reyes E.D., Mojica L., Dia V., Wang H., Wang T., Johnson L.A. (2021). Potential health benefits associated with lunasin concentration in dietary supplements and lunasin-enriched soy extract. Nutrients.

[17] Shidal C., Inaba J.I., Yaddanapudi K., Davis K.R. (2017). The soy-derived peptide lunasin inhibits invasive potential of melanoma initiating cells. Oncotarget.

[18] Galvez A.F., Chen N., Macasieb J., de Lumen B.O. (2001). Chemopreventive property of a soybean peptide (lunasin) that binds to deacetylated histones and inhibits acetylation. Cancer Res..

[19] Hsieh C.-C., Hernández-Ledesma B., de Lumen B.O., Victor R.P., Ronald Ross W. (2020). Cancer chemopreventive potential of seed proteins and peptides. Nuts and Seeds in Health and Disease Prevention.

[20] Sánchez-Quesada C., López-Biedma A., Toledo E., Gaforio J.J. (2018). Squalene Stimulates a Key Innate Immune Cell to Foster Wound Healing and Tissue Repair. Evid. Based Complement. Altern. Med..

[21] Barone A., Mendes M., Cabral C., Mare R., Paolino D., Vitorino C. (2019). Hybrid nanostructured films for topical administration of simvastatin as coadjuvant Treatment of melanoma. J. Pharm. Sci..

[22] Kang J.H., Jang W.Y., Ko Y.T. (2017). The effect of surface charges on the cellular uptake of liposomes investigated by live cell imaging. Pharm. Res..

[23] Song W., Bossy B., Martin O.J., Hicks A., Lubitz S., Knott A.B., Bossy-Wetzel E. (2008). Assessing mitochondrial morphology and dynamics using fluorescence wide-field microscopy and 3D image processing. Methods.

[24] Dakup P.P., Porter K.I., Little A.A., Zhang H., Gaddameedhi S. (2020). Sex differences in the association between tumor growth and T cell response in a melanoma mouse model. Cancer Immunol. Immunother..

[25] Lee Y.S., Jung Y.Y., Park M.H., Yeo I.J., Im H.S., Nam K.T., Kim H.D., Kang S.K., Song J.K., Kim Y.R. (2018). Deficiency of parkin suppresses melanoma tumor development and metastasis through inhibition of MFN2 ubiquitination. Cancer Lett..

[26] Crowe A., Yue W. (2019). Semi-quantitative determination of protein expression using immunohistochemistry staining and analysis: An integrated protocol. Bio-Protocol.

[27] Babak M.V., Chong K.R., Rapta P., Zannikou M., Tang H.M., Reichert L., Chang M.R., Kushnarev V., Heffeter P., Meier-Menches S.M. (2021). Interfering with metabolic profile of triple-negative breast cancers using rationally designed metformin prodrugs. Angew. Chem. Int. Ed..

[28] Bankhead P., Loughrey M.B., Fernández J.A., Dombrowski Y., McArt D.G., Dunne P.D., McQuaid S., Gray R.T., Murray L.J., Coleman H.G. (2017). QuPath: Open source software for digital pathology image analysis. Sci. Rep..

[29] Smith S.M., Baker M., Halebian M., Smith C.J. (2017). Weak molecular interactions in clathrin-mediated endocytosis. Front. Mol. Biosci..

[30] Francia V., Reker-Smit C., Boel G., Salvati A. (2019). Limits and challenges in using transport inhibitors to characterize how nano-sized drug carriers enter cells. Nanomedicine.

[31] Chang C.C., Wu M., Yuan F. (2014). Role of specific endocytic pathways in electrotransfection of cells. Mol. Ther. Methods Clin. Dev..

[32] Dutta D., Donaldson J.G. (2012). Search for inhibitors of endocytosis. Cell. Logist..

[33] Hillaireau H., Couvreur P. (2009). Nanocarriers’ entry into the cell: Relevance to drug delivery. Cell. Mol. Life Sci..

[34] Ojeda E., Puras G., Agirre M., Zarate J., Grijalvo S., Eritja R., Digiacomo L., Caracciolo G., Pedraz J.L. (2016). The role of helper lipids in the intracellular disposition and transfection efficiency of niosome formulations for gene delivery to retinal pigment epithelial cells. Int. J. Pharm..

[35] Trung Bui D., Nicolas J., Maksimenko A., Desmaële D., Couvreur P. (2014). Multifunctional squalene-based prodrug nanoparticles for targeted cancer therapy. Chem. Commun..

[36] Abrunhosa F., Faria S., Gomes P., Tomaz I., Pessoa J.C., Andreu D., Bastos M. (2005). Interaction and lipid-induced conformation of two cecropin−melittin hybrid peptides depend on peptide and membrane composition. J. Phys. Chem. B.

[37] Saleh J. (2018). Murine models of melanoma. Pathol. Res. Pract..

[38] Xia H., Tang Y., Huang R., Liang J., Ma S., Chen D., Feng Y., Lei Y., Zhang Q., Yang Y. (2022). Nanoliposome use to improve the stability of phenylethyl resorcinol and serve as a skin penetration enhancer for skin whitening. Coatings.

[39] Verma A., Najahi-Missaoui W., Cummings B.S., Somanath P.R. (2020). Sterically stabilized liposomes targeting P21 (RAC1) activated kinase-1 and secreted phospholipase A_2_ suppress prostate cancer growth and metastasis. Oncol. Lett..

[40] Barati M., Mirzavi F., Nikpoor A.R., Sankian M., Namdar Ahmadabad H., Soleimani A., Mashreghi M., Tavakol Afshar J., Mohammadi M., Jaafari M.R. (2021). Enhanced antitumor immune response in melanoma tumor model by anti-PD-1 small interference RNA encapsulated in nanoliposomes. Cancer Gene Ther..

[41] König S., Regen T., Dittmann K., Engelke M., Wienands J., Schwendener R., Hanisch U.K., Pukrop T., Hahn H. (2013). Empty liposomes induce antitumoral effects associated with macrophage responses distinct from those of the TLR1/2 agonist Pam3CSK 4 (BLP). Cancer Immunol. Immunother..

[42] Graeser R., Bornmann C., Esser N., Ziroli V., Jantscheff P., Unger C., Hopt U.T., Schaechtele C., Von Dobschuetz E., Massing U. (2009). Antimetastatic effects of liposomal gemcitabine and empty liposomes in an orthotopic mouse model of pancreatic cancer. Pancreas.

[43] Jose A., Labala S., Ninave K.M., Gade S.K., Venuganti V.V.K. (2018). Effective skin cancer treatment by topical co-delivery of curcumin and STAT3 siRNA using cationic liposomes. AAPS PharmSciTech.

[44] Petrilli R., Eloy J.O., Saggioro F.P., Chesca D.L., de Souza M.C., Dias M.V.S., da Silva L.L.P., Lee R.J., Lopez R.F.V. (2018). Skin cancer treatment effectiveness is improved by iontophoresis of EGFR-targeted liposomes containing 5-FU compared with subcutaneous injection. J. Control. Release.

[45] Singh A.K. (2016). Chapter 6- Nanoparticle pharmacokinetics and toxicokinetics. Engineered Nanoparticles.

[46] Wang Y., Marshall K.L., Baba Y., Lumpkin E.A., Gerling G.J. (2015). Compressive viscoelasticity of freshly excised mouse skin is dependent on specimen thickness, strain level and rate. PLoS ONE.

[47] Gómez-Valenzuela F., Escobar E., Pérez-Tomás R., Montecinos V.P. (2021). The inflammatory profile of the tumor microenvironment, orchestrated by cyclooxygenase-2, promotes epithelial-mesenchymal transition. Front. Oncol..

[48] Folkman J. (2002). Role of angiogenesis in tumor growth and metastasis. Semin. Oncol..

[49] Riemma-Pierre M.B., dos Santos Miranda Costa I. (2011). Liposomal systems as drug delivery vehicles for dermal and transdermal applications. Arch. Dermatol. Res..

[50] Moretti S., Spallanzani A., Chiarugi A., Fabiani M., Pinzi C. (2001). Correlation of Ki-67 expression in cutaneous primary melanoma with prognosis in a prospective study: Different correlation according to thickness. J. Am. Acad. Dermatol..

[51] Nagarajan P., Tetzlaff M.T., Curry J.L., Prieto V.G. (2017). Use of new techniques in addition to IHC applied to the diagnosis of melanocytic lesions, with emphasis on CGH, FISH, and mass spectrometry. Actas Dermosifiliogr..

[52] Kim H.Y., Jung H., Kim H.M., Jeong H.J. (2021). Surfactin exerts an anti-cancer effect through inducing allergic reactions in melanoma skin cancer. Int. Immunopharmacol..

[53] Gonzalez de Mejia E., Wang W., Dia V.P. (2010). Lunasin, with an arginine-glycine-aspartic acid motif, causes apoptosis to L1210 leukemia cells by activation of caspase-3. Mol. Nutr. Food Res..

[54] Xu P., Ning P., Wang J., Qin Y., Liang F., Cheng Y. (2019). Precise control of apoptosis via gold nanostars for dose dependent photothermal therapy of melanoma. J. Mater. Chem. B.

[55] Nascimento F.R., Viktor de Paula Barros Baeta J., Prado de França A.A., Braga Rocha e Oliveira M.A., Pizziolo V.R., Aparecida dos Santos A., Antônio de Oliveira Mendes T., Diaz-Muñoz G., Nogueira Diaz M.A. (2022). Dibenzoylmethane derivative inhibits melanoma cancer in vitro and in vivo through induction of intrinsic and extrinsic apoptotic pathways. Chem. Biol. Interact..

[56] Donato A.L., Huang Q., Liu X., Li F., Zimmerman M.A., Li C.Y. (2014). Caspase 3 promotes surviving melanoma tumor cell Growth after cytotoxic therapy. J. Invest. Dermatol..

[57] D’Arcy M.S. (2019). Cell death: A review of the major forms of apoptosis, necrosis and autophagy. Cell Biol. Int..

[58] Jacobs K.M., Bhave S.R., Ferraro D.J., Jaboin J.J., Hallahan D.E., Thotala D. (2012). GSK-3β: A bifunctional role in cell death pathways. Int. J. Cell Biol..

[59] Zhang C., Liu S., Yuan X., Hu Z., Li H., Wu M., Yuan J., Zhao Z., Su J., Wang X. (2016). Valproic acid promotes human glioma U87 cells apoptosis and inhibits glycogen synthase kinase-3β through ERK/Akts signaling. Cell. Physiol. Biochem..

[60] Atkinson J.M., Rank K.B., Zeng Y., Capen A., Yadav V., Manro J.R., Engler T.A., Chedid M. (2015). Activating the Wnt/β-catenin pathway for the treatment of melanoma—Application of LY2090314, a novel selective inhibitor of glycogen synthase kinase-3. PLoS ONE.

[61] Piva M.B.R., Jakubzig B., Bendas G. (2017). Integrin activation contributes to lower cisplatin sensitivity in MV3 melanoma cells by inducing the Wnt signalling pathway. Cancers.

[62] Bachmann I.M., Ladstein R.G., Straume O., Naumov G.N., Akslen L.A. (2008). Tumor necrosis is associated with increased alphavbeta3 integrin expression and poor prognosis in nodular cutaneous melanomas. BMC Cancer.

[63] Ladstein R.G., Bachmann I.M., Straume O., Akslen L.A. (2012). Tumor necrosis is a prognostic factor in thick cutaneous melanoma. Am. J. Surg. Pathol..

[64] Hugdahl E., Bachmann I.M., Schuster C., Ladstein R.G., Akslen L.A. (2019). Prognostic value of uPAR expression and angiogenesis in primary and metastatic melanoma. PLoS ONE.

[65] Karsch-Bluman A., Feiglin A., Arbib E., Stern T., Shoval H., Schwob O., Berger M., Benny O. (2018). Tissue necrosis and its role in cancer progression. Oncogene.

[66] De Araújo Farias V., O’Valle F., Lerma B.A., de Almodóvar C.R., López-Peñalver J.J., Nieto A., Santos A., Fernández B.I., Guerra-Librero A., Ruiz-Ruiz M.C. (2015). Human mesenchymal stem cells enhance the systemic effects of radiotherapy. Oncotarget.

