# Supplementary material for: Liposomes Loaded with Amaranth Unsaponifiable Matter and Soybean Lunasin Prevented Melanoma Tumor Development Overexpressing Caspase-3 in an In Vivo Model
Source: Pharmaceutics. 2022 Oct 18;14(10):2214. doi: 10.3390/pharmaceutics14102214 (PMC9609684; doi:10.3390/pharmaceutics14102214)
Supplement: Supplementary file 1 [file pharmaceutics-14-02214-s001.zip › pharmaceutics-1936058-supplementary.pdf]

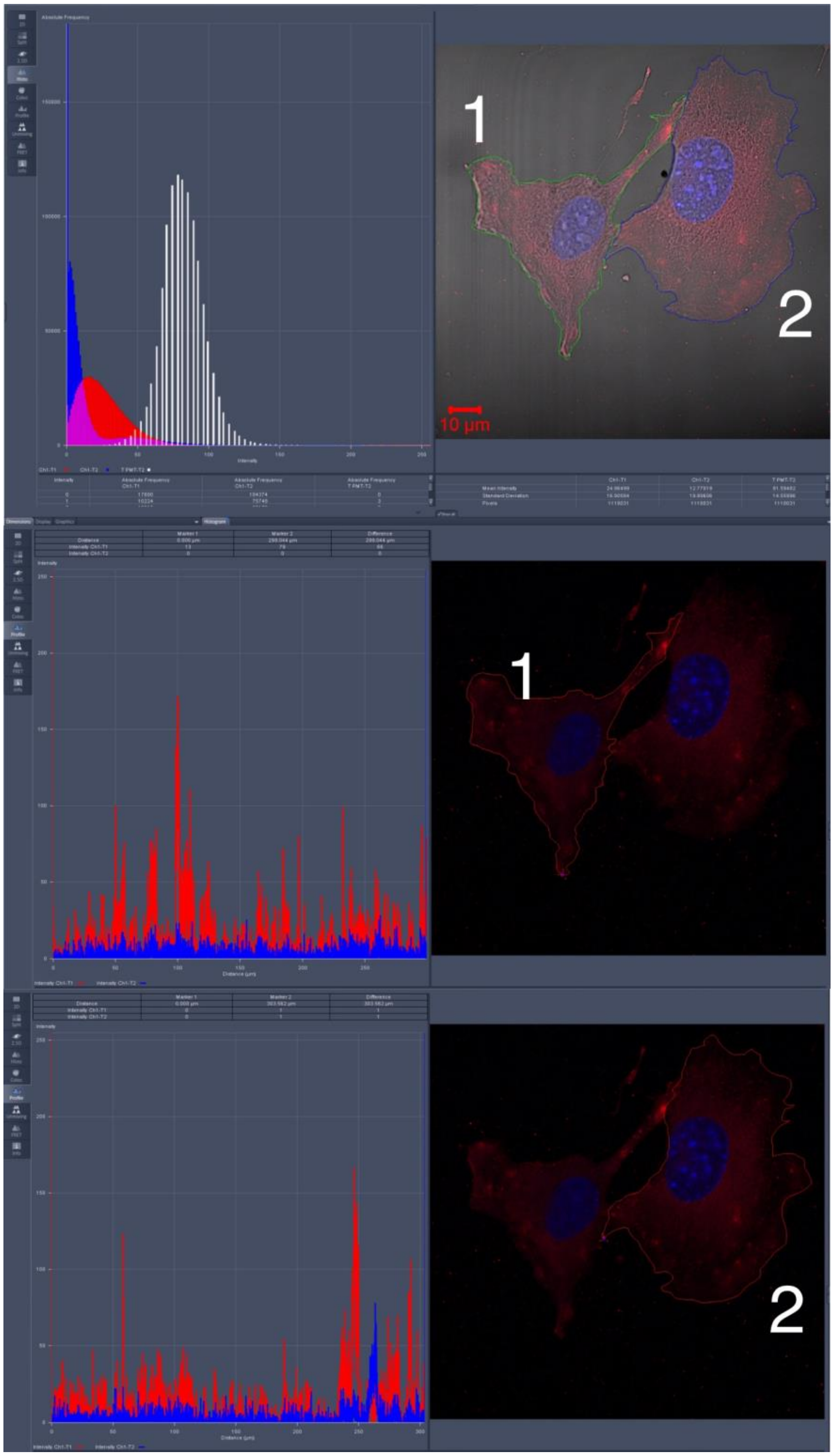

Figure S1. Intensity analysis using the Zeiss Zen Black software for the cell uptake of liposomes. To prevent quantifying the liposomes that are outside of the cells, the cell membrane edge was drawn manually. In this example, the analysis of two cells (numbers 1 and 2) are shown, as well as the intensity of the Hoechst 33342 (blue channel) and Rhodamine (red channel). The red channel was considered for the analysis.

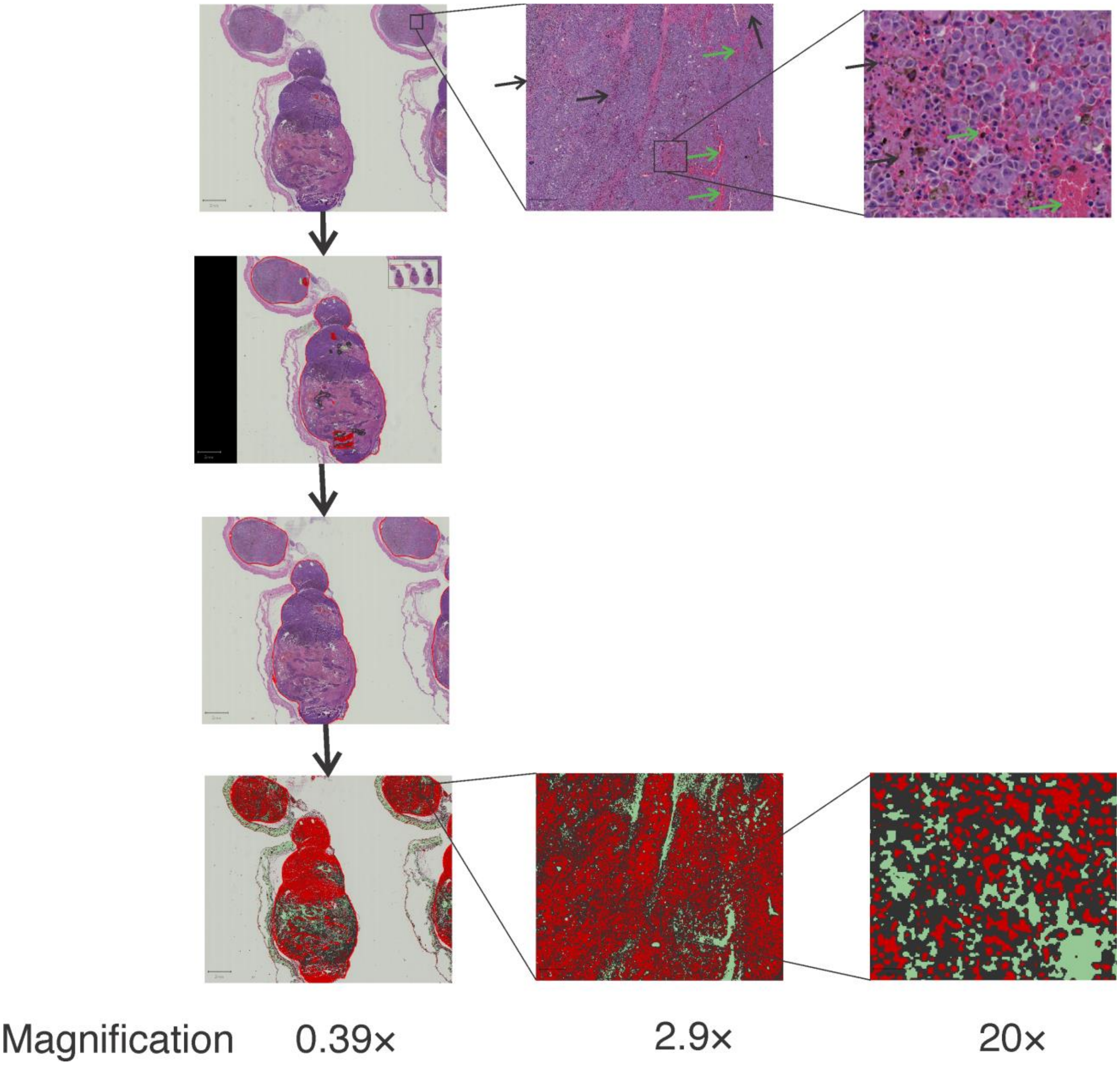

Figure S2. QuPath analysis for necrotic determination. In magnification 0.39× can be shown the workflow for setting up the training pixel classifier. With the wand tool several areas for necrotic (black arrows and shadows), viable cells (red shadows), and stroma (green arrows and shadows) should be set. Once the pixel classification was trained properly, the tumor edges should be selected and the different pixels for necrosis, viable cells and stroma should be measured. In magnification 2.9× and 20× can be shown the pixel classification in better detail. Black arrows and shadows show necrotic areas, green arrows and shadows show stroma, and red shadows show viable cells. The tumor belongs to G3 and it was just an example of the analysis process that was done in all the considered images in all the groups with tumor development (G3-G9) presented in figure 6.
